# Native Aortic Arch Interruption in Adulthood: Surgical Management

**DOI:** 10.1055/s-0039-1688957

**Published:** 2019-10-15

**Authors:** Duccio Federici, Samuele Bichi, Gianfranco Montesi, David Matiashvili, Cristina Agostinis, Cesare Morzenti, Sandro Sironi, Lorenzo Galletti

**Affiliations:** 1 Pediatric Cardiac Surgery, Ospedale del Cuore G. Pasquinucci, Monasterio Foundation, Massa, Italy; 2 Cardiac Surgery Unit, Department of Cardiovascular, “Papa Giovanni XXIII” Hospital, Bergamo, Italy; 3 Cardiac Surgery Unit, Department of Cardiothoracic and Vascular Surgery, “Santa Maria alle Scotte” Hospital, Siena, Italy; 4 Departement of Radiology, “Papa Giovanni XXIII” Hospital, Bergamo, Italy; 5 Pediatric Cardiac Surgery, Ospedale Pediatrico Bambin Gesù, Roma

**Keywords:** aortic arch interruption, complex aortic coarctations

## Abstract

Aortic coarctations in adults are mainly represented by recurrent critical narrowing at the site of previous surgical correction, or less frequently by native forms of complex obstructive malformations of the distal arch and isthmus. We present our experience with an unusual form of native adult aortic coarctation presenting as a complete interruption of the aortic arch.

## Case Presentation

**Fig. 1:**
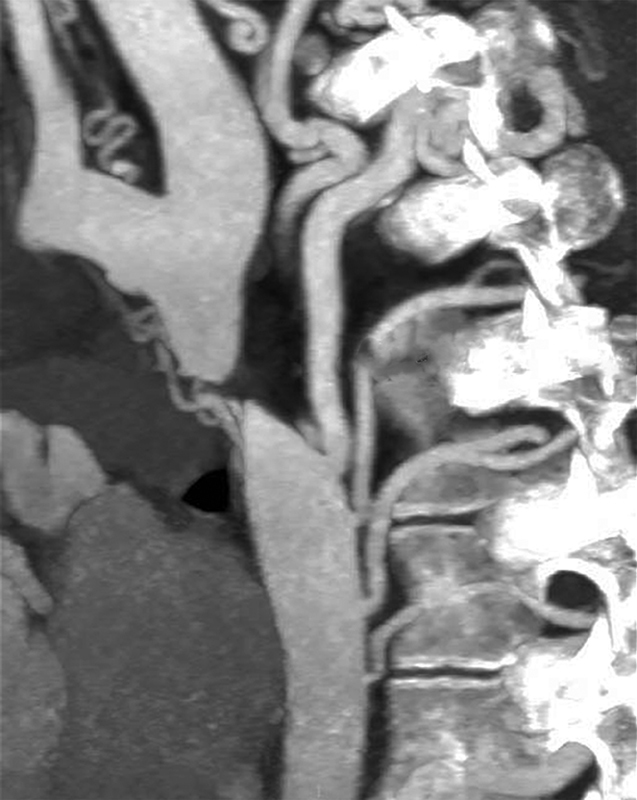
Preoperative computed tomography scan showing complete aortic arch interruption below the left subclavian artery with extensive arterial collateralization.

**Fig. 2:**
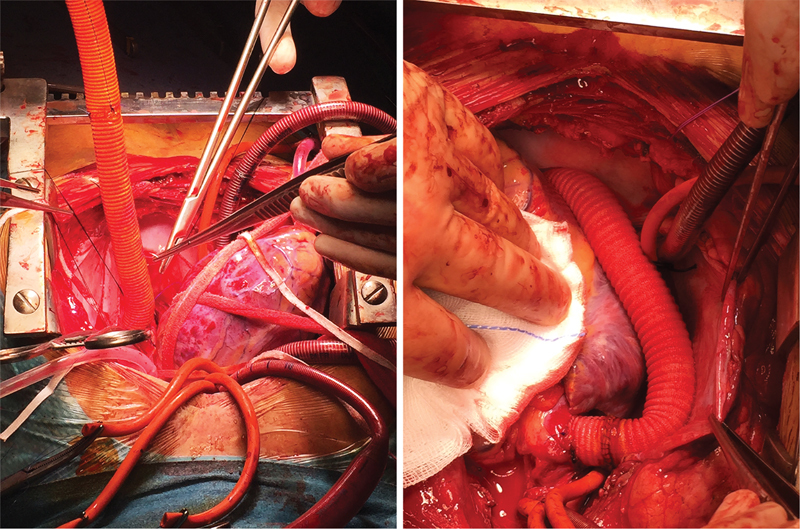
Intraoperative view of extra-anatomic ascending–descending aortic bypass performed under cardiopulmonary bypass.

**Fig. 3:**
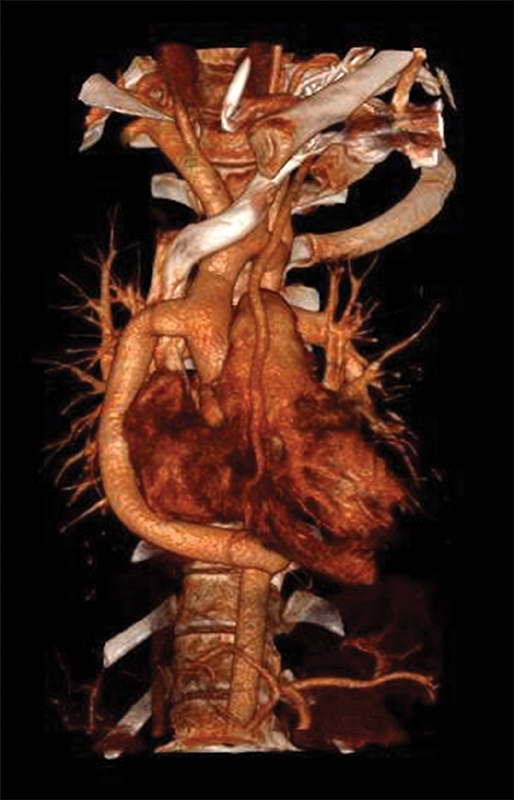
Postoperative computed tomography scan showing patent extra-anatomic conduit coursing along the right heart border.

An 18-year-old patient was referred to our institution with the diagnosis of native interruption of the aortic arch. The clinical picture was characterized by severe hypertension of the upper extremities with bilateral noninvasive brachial pressure values around 170/100 mm Hg. Femoral pulsatility was absent. Preoperative computed tomography (CT) scan showed complete aortic interruption below the left subclavian artery with extensive arterial collateralization ( Fig. 1 ). Echocardiographic evaluation revealed a bicuspid well-functioning aortic valve and only a mild degree of left ventricular hypertrophy. Preoperative cerebral magnetic resonance imaging did not reveal any anomaly of the intracranial circle. The patient underwent extra-anatomic ascending-to-descending aortic bypass through median sternotomy ( Fig. 2 ). The procedure was performed under cardiopulmonary bypass on the beating heart to avoid hemodynamic instability during the distal and more challenging aortic anastomosis. 1 2 The descending thoracic aorta was exposed by opening the posterior pericardium longitudinally: particular care was taken in separating the aorta from the esophagus. After the distal anastomosis was accomplished, the posterior pericardium was closed between the aorta and the esophagus. The meaning of this maneuver is to separate and protect the esophagus from the aortic anastomosis. The postoperative course was characterized by rapid normalization of upper systemic pressure along with progressive recovery of femoral pulsatility. Postoperative CT scan showed a patent conduit coursing along the right heart border ( Fig. 3 ).

## Discussion

Aortic coarctations in adults are mainly represented by recurrent critical narrowing at the site of previous surgical correction or, less frequently, by native forms of complex obstructive malformations of the distal arch and isthmus. Our case is a very unusual form of native complex coarctation presenting as complete interruption of the aortic arch.

The extensive and impressive arterial collateralization around the interruption site, along with a thin aortic wall and poorly mobilizable descending aorta, are usually present in this pathology. These factors made direct aortic replacement challenging and dangerous in terms of bleeding risk.

For these reasons, we decided to treat the patient by means of extra-anatomic aortic bypass. In this way, we were able to relieve the obstruction while working far from the coarctation site. Our experience confirms the safety and effectiveness of this strategy for management of complex aortic coarctations in adults. 3
